# Production of Bitumen from Fuel Oil and Its Fractions

**DOI:** 10.3390/ma19081590

**Published:** 2026-04-15

**Authors:** Saule Bukanova, Gulbarshin Shambilova, Fazilat Kairliyeva, Aigul Bukanova, Nagima Karabassova, Abzal Taltenov, Igor Makarov, Ivan Levin, Georgy Makarov, Junlong Song

**Affiliations:** 1 Institute of Petrochemical Engineering and Ecology Named After N.K. Nadirov, Atyrau University of Oil and Gas Named After Safi Utebayev, Atyrau 060027, Kazakhstan; kairlieva.fazi@mail.ru (F.K.); bukanova66@mail.ru (A.B.); nagima@inbox.ru (N.K.); 2 Department of Chemistry and Chemical Technology, Kh. Dosmukhamedov Atyrau University, Atyrau 060011, Kazakhstan; 3 Department of Chemistry, L.N. Gumilyov Eurasian National University, 2 Satpayev Street, Astana 010000, Kazakhstan; abzal06@mail.ru; 4 A.V. Topchiev Institute of Petrochemical Synthesis, Russian Academy of Sciences, 29 Leninsky Prospect, Moscow 119991, Russia; makarov@ips.ac.ru (I.M.);; 5 Faculty of Chemistry, Lomonosov Moscow State University, GSP-1, 1-3 Leninskiye Gory, Moscow 119991, Russia; georgii.makarov@chemistry.msu.ru; 6 International Innovation Center for Forest Chemicals and Materials, Nanjing Forestry University, Nanjing 210037, China; junlong.song@njfu.edu.cn

**Keywords:** road bitumen, sampling depth, oxidation of fuel oil, penetration, ductility, brittleness, conditional viscosity

## Abstract

This paper examines the effect of gas oil fraction extraction depth from fuel oil on the physicochemical and performance properties of road bitumen. The study’s novelty lies in establishing the relationship between the seven-component chemical group composition of heavy residues and their oxidation kinetics. It has been experimentally demonstrated that using feedstock with a nominal viscosity (VU80) in the range of 20–80 s (corresponding to fractions of 480–525 °C) enables the production of bitumen that simultaneously meets the requirements of ASTM D946, EN 12591, and ST RK 1373. The paper substantiates an optimal “viscosity range” for processing non-standard feedstock, ensuring increased resistance of the finished product to thermal-oxidative aging.

## 1. Introduction

Currently, the following technologies are mainly used to produce road bitumen [1,2]:

- Direct oxidation of oil residues (tar) and mixed compositions of tar with other residual products of oil refining [3,4];
- Deep vacuum distillation of fuel oil from high-sulfur, high-tar oils (residual bitumen) [5];
- Compounding of propane-butane or butane de-asphalting asphalts with oil residues of various origins [6];
- Compounding of high-melting bitumen with feedstock, other residues, plasticizing additives and wastes [7,8,9].

The most common bitumen production technology consists of direct oxidation of oil residues (tar) and mixed tar compositions with other residual products of oil refining.

Oxidized bitumen can be semi-liquid, viscous, and relatively solid. Oxidation (hot air purging) is used when the feedstock contains an insufficient amount of asphalt-resin substances. Air purging allows their content in the bitumen composition to be increased to the required level [10,11]. Oxidized petroleum bitumen was first produced on an industrial scale as early as 1844 (at the suggestion of J.G. Bierley) by bubbling air through a layer of oil residues at 204 and 316 °C [12].

The process of raw material oxidation to bitumen [13] is a set of heterogeneous reactions occurring on the surface of gas and liquid reacting media. When purging raw materials with air, the content of resins and asphaltenes increases, and the content of oils decreases. The relative content of paraffin-naphthenic and aromatic compounds decreases with deepening oxidation, the asphaltene content increases, which leads to an increase in the softening temperature of bitumen, as well as a decrease in ITS penetration and ductility [14].

Oil residue oxidation processes using air are carried out in batch or continuous plants. Reaction apparatuses of various types are used for this purpose: cubes (periodic and continuous), tubular and non-compressor reactors, and various designs of barbotage-type reactors.

In recent years, hollow oxidation columns have been widely used as reactors for continuous operation bitumen plants [15].

Residual petroleum bitumen is obtained by concentrating oil residues through deep vacuum distillation of straight-run fuel oils sourced from the atmospheric distillation of highly viscous, high-sulfur, high-resinous oils. Sulfurous and low-sulfur oils are practically unsuitable for the production of residual road bitumen with regulatory characteristics. The remnants of deep vacuum distillation of fuel oil from a commercial mixture of such oils with a given heat resistance are characterized by insufficient ductility and unsatisfactory low-temperature characteristics.

The yield of residual bitumen during deep vacuum distillation of highly viscous, highly resinous fuel oils depends primarily on the content and composition of asphalt-resinous substances, as well as on the nature of the oil and its group chemical composition [16].

During vacuum distillation, the content of paraffin-naphthenic, monocyclic and bicyclic aromatic compounds in the residue decreases. The content of aromatic polycyclic hydrocarbons, resins and asphaltenes increases.

The temperature of the raw material (fuel oil) at the outlet of the tubular furnace and at the entrance to the vacuum column should not exceed 400–420 °C in order to avoid its thermal decomposition and the formation of coke in the contact devices of the column. The fuel oil residence time in the furnace should be minimal. The residual pressure during deep vacuum distillation depends on the nature of the residue and the specified properties of the residual product. Usually, the pressure in the supply zone of the vacuum column is in the range of 4.65–9.30 kPa, and in the upper part, about 2.50 kPa. As the vacuum depth and process temperature increase, the softening temperature of the residue increases, and the penetration decreases.

To increase the plasticity and frost resistance of the residual bitumen, vacuum distillation is continued until the “softening temperature” of the residue is slightly higher than the required value, after which this indicator is adjusted to the norm by diluting the lower side straps or other less viscous residues [17].

A number of enterprises do not have vacuum units at primary oil refining plants. Then, the vacuum distillation of fuel oil is carried out directly at bitumen oxidation plants in order to provide the oxidation capacities with bitumen raw materials—tar. In this case, a wide gas oil fraction is usually removed from the vacuum column, which is then used as a raw material in secondary refining processes.

Each individual raw material requires an individual approach to its processing modes; therefore, the design solutions of vacuum blocks, vacuum generating system types, types of plates and nozzles can be diverse, designed to solve specific technological problems of a particular enterprise.

The basis for the production of non-oxidized compounded bitumen road is usually the products of de-asphalting tar processes with propane solvents. The main purpose of propane tar de-asphalting is to obtain deasphaltisates, which are raw materials for the production of base oils, as well as raw materials for the processes of secondary oil refining—catalytic cracking and hydrocracking. The remnants of de-asphalting (asphalt, asphaltene) in some cases meet the requirements for binders used in road construction [18,19]. However, more often these residues are used as a raw component of bitumen feedstock for its subsequent oxidation in a mixture with tar.

High-quality non-oxidized compounded road bitumen can be obtained on the basis of propane (propane-butane, butane) de-asphalting asphalts [20,21]. Weighted asphalts, which are concentrates of resins and asphaltenes, serve as the structural framework of bitumen. By compounding such asphalt with specially selected plasticizing components, it is possible to obtain road bitumen of a fairly wide range and high quality [22]. Raw tar, extracts of selective oil purification, heavy side straps removed from vacuum columns and other components are usually used as plasticizers, based on the characteristics of the processed raw materials and their composition and the technology of each specific production.

It should be noted that using this technology, it is possible to obtain conditioned road bitumen from highly paraffinic oils [23], since paraffins and most of the paraffin-naphthenic hydrocarbons pass into deasphaltisate.

In recent years, there has been a noticeable increase in the production of petroleum fuel components. Lightweight components are additionally distilled from the tar. The resulting weighted tar has a viscosity of 3–10 times higher than the current regulatory values.

An effective approach to obtaining high-grade road binders from petroleum residues with abnormal viscosity characteristics is the use of a combined “overoxidation → dilution” scheme [24,25].

The essence of the technology is the deep oxidation of feedstock with any viscosity in the range of 20–200 (or more) seconds. This creates a kind of structural framework of bitumen, characterized by a high content of resins and asphaltenes. The resulting over-oxidized bitumen is then plasticized via the addition of non-oxidized plasticizing components, if necessary [26].

Unlike direct oxidation, where viscosity increases with a deficiency of plasticizing components throughout the product, the compounding method achieves specified viscosity while maintaining a high content of unoxidized components, which determines the superior low-temperature properties of such bitumens.

Along with traditional methods, modern research is actively focused on improving the performance properties of road surfaces by creating modified binders, including the use of rubber crumb, polymer additives, and the development of emulsified asphalt systems. However, the effectiveness of such modifiers is largely determined by their physicochemical compatibility with the base bitumen. The primary objective remains the development of scientifically sound approaches to the production of stable oxidized bitumens that meet international standards.

In connection with the above, the present work was undertaken, the purpose of which was to compare the properties of bitumen obtained by oxidation of fuel oil and its fractions with different depths of gas oil extraction.

## 2. Materials and Methods

M-100 grade fuel oil (produced at Pavlodar Oil Refinery, Pavlodar, Kazakhstan) from the commercial mixture of West Siberian oils coming for processing was used as feedstock. General characteristics of the fuel oil used are presented in Table 1.

The fractional composition of fuel oil was determined by the following method: fuel oil (500 mL) was vacuum distilled at a residual pressure of no more than 26.6 Pa (0.2 mmHg). The heating was carried out at such a speed that the first drop fell into the vacuum successor 15–20 min after reaching the required vacuum. Thus, the heating of the flask during distillation was adjusted so that the distillation was carried out evenly at a rate of one drop per second throughout the experiment. To establish the temperature of the start of distillation, the temperature and residual pressure in the system were recorded at the moment when the second drop fell into the vacuum receiver. The temperature and residual pressure in the system were assumed to be the temperature of the end of boiling of the fuel oil at the moment when, due to the cessation of vapor release from the bulb (despite continued heating), the temperature begins to decrease. At this point, the bulb heating is turned off.

**Figure 1:**
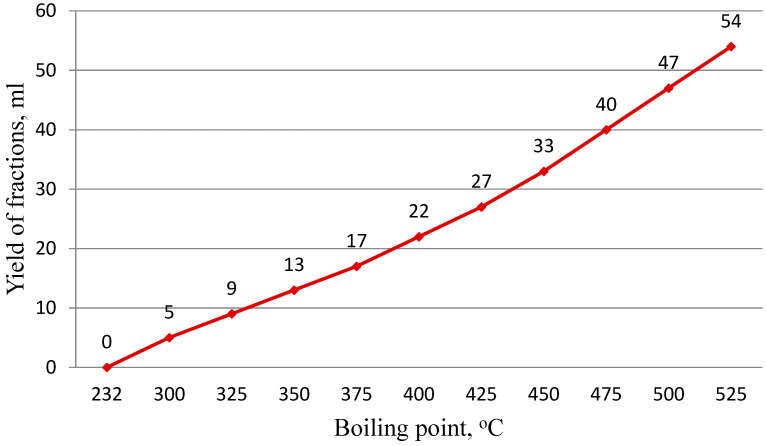
The curve of deep vacuum fuel oil dispersal.

The curve of deep vacuum fuel oil dispersal is shown in Figure 1.

The technology for obtaining bitumen samples is as follows: direct oxidation of fuel oil, as well as fractions obtained from this fuel oil distilled at 480 °C, 500 °C and 525 °C.

The oxidation conditions were identical throughout the entire series of experiments: process temperature was 240–260 °C; the air mixture was supplied for bubbling at a rate of 1.5 L/min per 1 kg of the initial product.

The oxidation of oil residues in the laboratory was carried out in a laboratory cube. In general, the process of oxidation of raw materials in the laboratory is similar to the industrial process of bitumen production in oxidation cubes.

**Figure 2:**
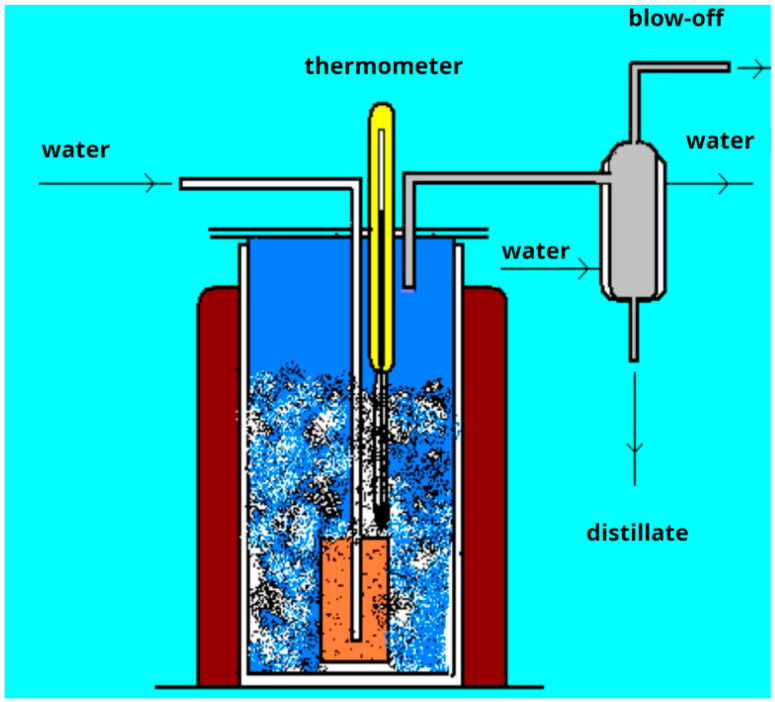
Laboratory oxidation cube.

The experiment was carried out in a laboratory thermally insulated apparatus with adjustable electric heating (Figure 2), which has a nozzle for air supply to oxidation, for the removal of distillation and oxidation gases. A preheated oil residue is poured into the device. The working volume of the product device should not exceed 70% of the geometric volume. When the product temperature reaches 140–150 °C, the air supply begins in the device. The flow rate of air supplied for oxidation is regulated by a rotameter.

The following methods were used to determine the characteristics of bitumen:

- The softening temperature (T_p_) is determined on a Ring and Ball device [27]. The essence of the method is to determine the temperature at which bitumen contained in a ring of specified dimensions softens under test conditions and, moving under the action of a steel ball, reaches the bottom plate.
- The brittleness temperature [28] is the temperature at which the material collapses under the action of a short-term applied load. The brittleness temperature characterizes the behavior of bitumen at low temperatures. The essence of the method is to cool and periodically bend a bitumen sample and determine the temperature at which cracks appear or the bitumen sample breaks.
- Penetration [29] characterizes the hardness of bitumen and is defined as the depth of immersion (penetration) of a calibrated needle into a bitumen sample under the action of a certain load for a given time at a fixed temperature.
- Ductility is the ability of bitumen to stretch into a thread. It is defined as the length of the thread formed at the moment of rupture at fixed loads and temperatures of 25 °C (D_25_), 0 °C (D_0_) [30].

The resulting residue from deep vacuum distillation of fuel oil has a softening temperature according to the “ring and ball” method [27] equal to 35.5 °C.

The group chemical composition of the residues was determined by liquid adsorption chromatography with gradient displacement for separation into seven groups: paraffin-naphthenic hydrocarbons (PNH), light (LAH), medium (MAH), heavy aromatic hydrocarbons (HAH), resins and asphaltenes.

## 3. Results and Discussion

The oxidation of fuel oil, carried out in compliance with the operating parameters, made it possible to obtain bitumen with the characteristics shown in Table 2.

The analysis shows that this bitumen does not meet the “Elongation” indicator specification established by the standards of Kazakhstan and ASTM [31].

Therefore, fractions (residues) boiling off above 480 °C, 500 °C and 525 °C were obtained and studied by deep vacuum distillation, which were subsequently subjected to oxidation. The main residue characteristics are given in Table 3.

All indicators of physical and mechanical properties were determined in accordance with the requirements of the relevant standards (ASTM, ST RK), while the final values were taken as the average results of parallel determinations performed within the limits of the standardized convergence of the methods.

As follows from the presented data, fuel oil fractions with an initial boiling point above 480 °C, 500 °C and 525 °C differ significantly in the concentrations of various groups of hydrocarbons. As the sampling depth increases, the content of paraffin-naphthenic hydrocarbons in the residues decreases, which are removed with gas oil fractions, and the content of aromatic compounds increases due to evaporation of light monocyclic hydrocarbons. As the sampling depth increases, the content of resins and asphaltenes increases naturally. Accordingly, the coking tendency of the residue increases.

As the sampling deepens, the content of paraffin-naphthenic, light and medium (mono– and bicyclic) aromatic compounds decreases in the residues, which are removed from the vacuum column with side straps—light and heavy vacuum gas oil. Due to this, the proportion of heavy polycyclic aromatic compounds increases.

The increase in weight of the oil residue, caused by the increasing depth of distillate extraction, leads to a natural accumulation of asphaltenes. Along with the change in group composition, an increase in viscosity (from 29.8 to 76.2 s at VU80 (°E)) and density (up to 1002 kg/m^3^) is observed. This, combined with the increase in sulfur concentration, leads to increased coking of the heavier residues.

When assessing the greatest suitability of the obtained residues for use as raw materials for the production of bitumen road grades according to the value of the indicator “conditional viscosity at 80 °C” [32], preference should be given to the residue boiling off above 480 °C, since the viscosity of such residue is in the range of 20–40 s, and the residue boiling off above 500 °C, since its viscosity corresponds to various grades of bitumen 85–100 and 120–150 [29] with a viscosity of 40–60 s. As for the residue boiling above 525 °C, its suitability or unsuitability for ensuring the production of high-quality commercial products should be separately proven experimentally, since the viscosity of the residue significantly exceeds the maximum standard values.

The high concentration of heavy aromatic hydrocarbons in the studied residues indicates their increased reactivity. It is known [33] that during oxidation, the heaviest portion of the aromatic compounds actively reacts and is consumed to form high-molecular polycyclic structures, which include resins and, ultimately, asphaltenes.

However, as the sampling deepens, the content of plasticizing components—oils—decreases in the residue, which negatively affects the low–temperature characteristics of bitumen and its plasticity.

Thus, the objective of the study is not to select a residue with a certain depth of selection of gas oil fractions of hydrocarbons, but to scientifically and practically determine the range of selection depths in which residues will be found that are the most favorable raw materials for obtaining highly effective binders.

**Figure 3:**
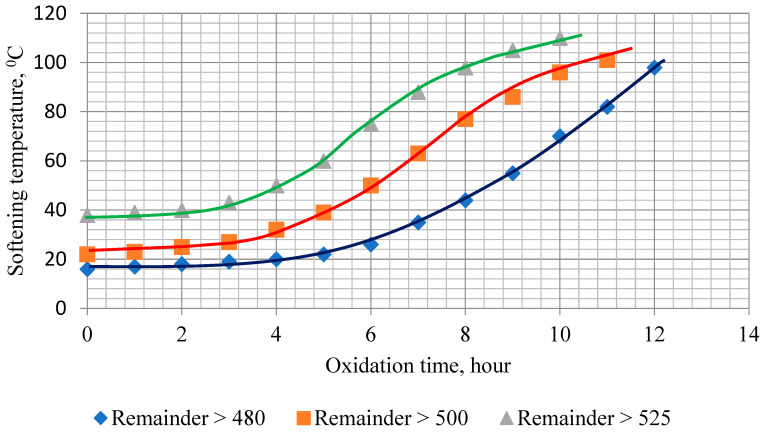
Kinetics of oxidation of West Siberian oil residues of different sampling depths.

The rate of change in softening temperature with oxidation time for residues with different sampling temperatures is shown in Figure 3.

The presented dependences indicate that heavier fuel oil distillation residues (fraction 525 °C) oxidize faster at the initial stage of the process than light residues. This is potentially because when a heavier residue is oxidized to the required degree of transformation, the increase in the concentration of resins and asphaltenes will be less than when a lighter residue (fraction 480 °C) is oxidized, in which the content of these components is lower. Accordingly, the time required for the growth of a smaller number of reaction products turns out to be shorter.

A large amount of reaction products must accumulate in the lighter residue (fraction 480 °C) in order for it to turn into bitumen with a given degree of oxidation. Therefore, the time required to achieve the required degree of transformation is longer (12 h).

The residue boiling above 525 °C has a minimum duration of the initial stage of slow structure formation and the highest oxidation rate until the T_p_ value is reached at about 100 °C. In the initial oxidation period (the first 7–8 h), the transformation reaction average rate constants have their highest values. However, in the future, as the most reactive components of the raw material mixture—aromatic compounds—are consumed, the reaction rate decreases, the rate of increase in softening temperature decreases markedly, and the process itself practically stops under these oxidation conditions after 9–10 h of oxidation. To continue the oxidation of this residue, it is necessary to increase in the process energy, for example, by increasing the reaction temperature.

The residue with the lowest extraction depth of gas oil fractions, boiling off above 480 °C, has a longer induction period and lower average reaction rates during the initial oxidation period (7–8 h) compared to the other samples. However, this residue is characterized by the highest content of aromatic compounds in its composition.

Of scientific interest is the analysis of the transformation of the hydrocarbon composition of bitumen obtained by thermal oxidation of fuel oil fractions with different temperature selection thresholds.

In the process of oil residue oxidation, many sequential and parallel reactions occur simultaneously, resulting in an increase in the molecular weight of the oxidized product. Chemical transformations of the raw material during air blowing are caused by the progressive elimination of hydrogen, which, combined with cyclization processes, initiates the synthesis of complex high-molecular structures. These transformations result in the intensive accumulation of asphaltene–resin compounds in the system, which act as the structural framework of the bitumen. Consequently, the chemical group compositions of the original residues and the bitumens obtained from them differ significantly.

When considering the obtained results, the following regularities and differences in chemical group compositions were found.

Bitumens are characterized by higher content of resins and asphaltenes, lower content of paraffinonaphthenic and aromatic hydrocarbons in comparison with initial residues.

**Figure 4:**
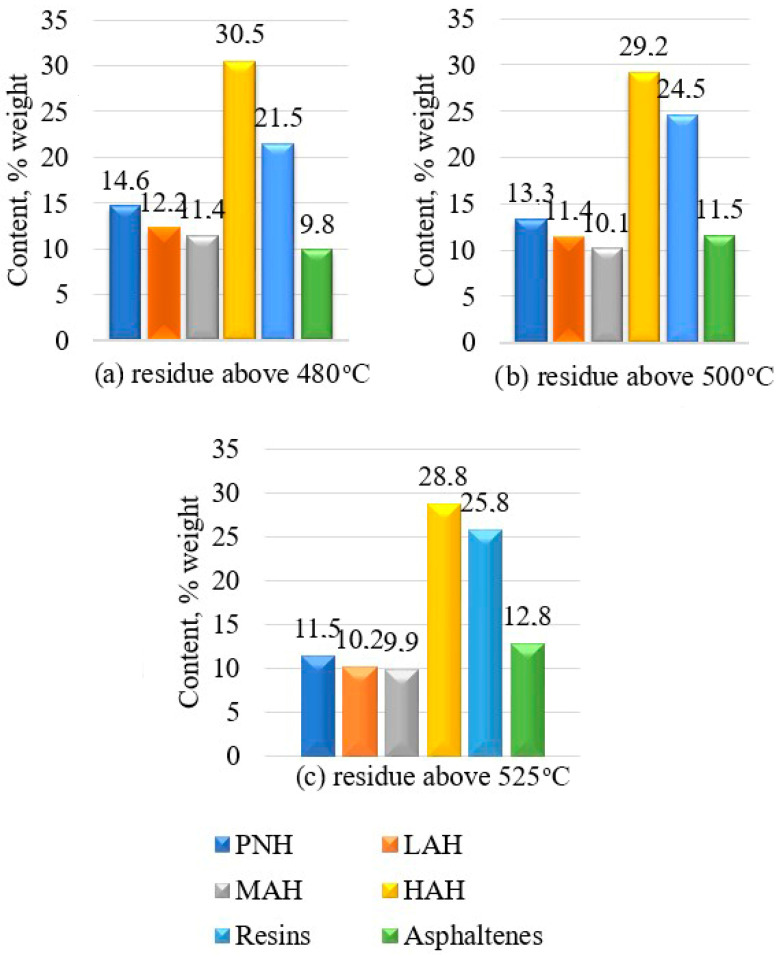
Group chemical compositions of binders obtained from petroleum residues boiling above 480 °C, 500 °C and 525 °C.

Figure 4 shows the compositions of group chemicals of bitumen obtained from residues of fuel oil distillation of different depths of sampling.

**Figure 5:**
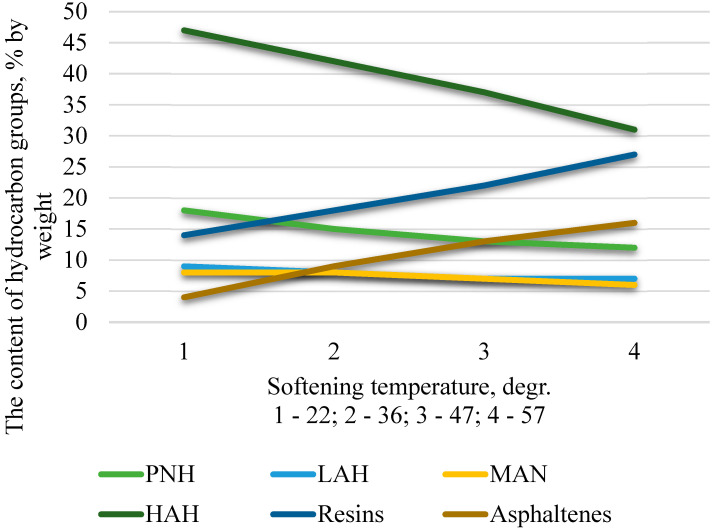
Dynamics of redistribution of hydrocarbon fractions during oxidative conversion of oil residues.

The nature of changes in individual groups of hydrocarbons during the oxidation process is shown in Figure 5.

A comparative analysis of the data presented in Figure 4 and Figure 5 indicates a significant reduction in the proportion of paraffin-naphthenic hydrocarbons in the finished bitumen relative to the feedstock. Considering the high chemical inertness of these compounds at the given temperature (240–260 °C), it can be concluded that their loss is not due to destructive transformations, but to the physical removal of the most volatile components along with the oxidation exhaust gases.

The resulting bitumen exhibits a consistent decrease in the concentration of light and medium aromatic hydrocarbons relative to their content in the feedstock. This trend is due not only to the physical removal of the most volatile fractions from the reaction zone along with the exhaust gases, but also to their active participation in chemical transformations. Heavier aromatic homologues act as reactants in oxidative polycondensation processes, transforming into high-molecular-weight polycyclic compounds—resins and asphaltenes—which have significantly higher viscosity and boiling points.

Heavy aromatic compounds in bitumen are the most reactive components of the raw material mixture, which enrich bitumen to the greatest extent with resinous compounds.

In turn, reactive resins form asphaltenes. From the experimental data presented, it follows that the content of resins and asphaltenes in bitumen significantly exceeds their content in the initial residues.

Note that the above quantitative relationships are valid only for cases of obtaining road bitumen. When the residues are oxidized to a more heat-resistant road bitumen, for example, the oxidation depth of the residues will be higher and, accordingly, the consumption of individual groups of aromatic hydrocarbons for transformations during oxidation will increase. Therefore, more oxidized bitumen will have a different composition of group chemicals, in which the content of aromatic compounds will be slightly lower than in the case of oxidation of the residue to the grade of road bitumen, and the content of resins and asphaltenes is higher. As a result of such redistribution of hydrocarbons in the bitumen composition, one should expect a deterioration in the deformation capacity of the binder, the degree of oxidation of which is higher. Then, the plasticity of bitumen with a reduced degree of oxidation will be higher and the low-temperature characteristics of such bitumen will be more preferable.

The main physio-chemical characteristics of road bitumen samples obtained by oxidation of residues of different sampling depths are presented in Table 4, which also presents the requirements of Kazakhstan, European and American standards for the corresponding grades of road bitumen.

The conducted comparison of the experimental characteristics of road binders obtained from fuel oil fractions with different sampling temperatures for compliance with the provisions of ST RK, ASTM and EN indicates that all the studied samples fully satisfy the regulatory quality criteria.

Thus, intensifying the process of extracting fractions from raw materials leads to a decrease in the deformation capacity of finished bitumen; however, samples based on the residue with a selection threshold of 480 °C remain the most plastic, despite the overall increase in ductility indicators.

**Figure 6:**
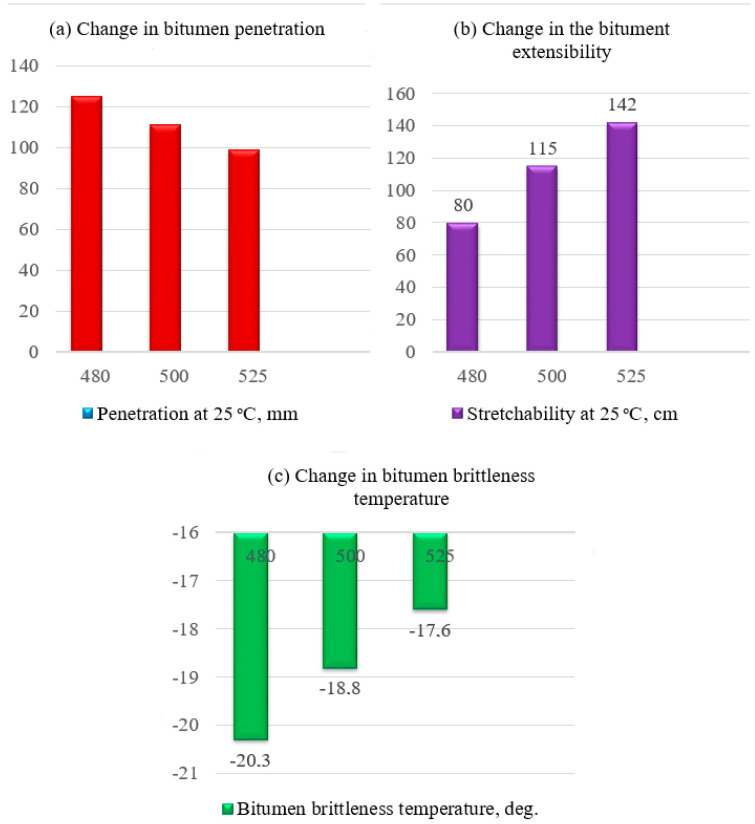
The nature of changes in bitumen properties depending on the depth of sampling of the initial residue.

At the same time, the low-temperature properties of bitumens deteriorate somewhat and are best for bitumens obtained from residues boiling above 480 °C. Moreover, all bitumens obtained by oxidizing fuel oil residues are characterized by good resistance to thermal-oxidative aging processes (Figure 6).

The decline in ductility of road binders with increasing gas oil extraction depth and rising bottoms temperature is due to a deficiency of paraffinic-naphthenic and aromatic compounds, which act as internal plasticizers. It is the retention of a significant proportion of these components in the residue boiling above 480 °C that determines its status as the most optimal raw material for producing bitumen with high deformation properties.

The improved low-temperature properties of bitumens synthesized from moderate-depth distillation residues directly correlate with the increased concentration of paraffin-naphthenic structures in their chemical profile. These components ensure the necessary mobility of the bitumen’s structural framework, preventing its premature transition to a brittle state at sub-zero temperatures.

As shown, if lighter residues are processed into bitumen, the “extensibility at 25 °C” parameter will be lower than the current standard, while other parameters meet the standard values. If heavier residues are processed, direct oxidation will produce bitumen with unsatisfactory low-temperature properties.

The results of the evaluation of the performance characteristics of the obtained bitumens are in strict accordance with the recorded dynamics of changes in their chemical structure, which emphasizes the decisive role of the composition of the original oil residue in the formation of the quality of the road binder.

Thus, the main technological requirement for bitumen’s raw materials produced from fuel oil is that the conditional viscosity at 80 °C must lie within a certain range; in the case under consideration, this range is 20–80 s.

## 4. Conclusions

It has been established that the optimal viscosity range for the production of road bitumen from fuel oil from the Pavlodar Oil Refinery is 20–80 s (according to VU80), which corresponds to the residues of the high-vacuum distillation of fuel oil, boiling in the range of 480–525 °C.

It has been experimentally confirmed that direct oxidation of such raw materials ensures the production of bitumen grades 70/100, 100/130, and 50/70, which fully meet the requirements of Kazakh, European, and American standards.

Based on a seven-group chemical analysis, it has been proven that the high oxidation rate of heavy residues (>525 °C) is due to the increased content of heavy aromatics (18.1%) and asphaltenes (45.9% in total), which form the structural basis of bitumen in the early stages of the process.

It was found that limiting the depth of selection at 525 °C (viscosity 76.2 s) is critical for maintaining low-temperature characteristics (brittleness temperature down to −20.4 °C), since further increase in weight leads to an irreversible deficiency of plasticizing components.

## Figures and Tables

**Table 1:** Characteristics of M-100 grade fuel oil.

Indices	Characteristics
Density, kg/m^3^	971.2
Specific viscosity, VS_80_, s	5.2
Flash point, °C	180
Group chemical composition *, wt%	
paraffin-naphthenic	23.0
light aromatic hydrocarbons	16.3
medium aromatic hydrocarbons	10.0
heavy aromatic hydrocarbons	34.1
resins I	6.8
resins II	7.7
asphaltenes	2.1

* The group chemical composition was determined by liquid chromatography on silica gel using the Gradient-M device.

**Table 2:** Characteristics of bitumen obtained by oxidation of fuel oil.

No.	Description of Indicators, Units of Measurement	Bitumen Obtained by Oxidation of Fuel Oil	Kazakhstan Standard (Grade 70/100)	ASTMD 946-82 [29]
1	Softening point, °C	50.5	not lower than 45	not normalized
2	Needle penetration depth at 25 °C, mm at 0 °C, mm			
		91 44	71–100 not less than 22	85–100 -
3	Elongation at 25 °C, cm at 0 °C, cm	19	not less 75	not less 100
		3.0	3.8	-
4	Brittleness temperature, °C	−23.2	not higher −20	-

**Table 3:** Physicochemical parameters of fuel oil fractions with temperature cutoff thresholds of 480 °C, 500 °C and 525 °C.

Description of Indicators, Units of Measurement	Source Fuel Oil	The Values of Indicators for Residues Boiling off Are Higher, °C		
		480	500	525
1. Relative density at 20 °C, kg/m^3^	971	994	999	1002
2. Flash point, °C	162	310	above 320	
3. Conditional viscosity at 80 °C, seconds	5.2	29.8	56.2	76.2
4. Softening point, °C	-	-	24.3	35.5
5. Group chemical composition: hydrocarbon content, % by weight - paraffin-naphthenic - aromatic, including - light - average - heavy - resin - asphaltenes	23.0 60.4 16.3 10.0 34.1 14.5 2.1	16.6 62.9 17.1 9.2 36.6 17.3 3.2	12.5 65.5 16.3 9.0 39.2 18.0 3.5	11.0 67.6 18.1 6.9 41.6 10.2 4.3

**Table 4:** Physicochemical characteristics of experimental binders obtained from the residues of the distillation of fuel oil from Pavlodar Oil Chemistry Plant, boiling in the range from 480 °C to 525 °C and the requirements of current standards for road bitumen.

Description of Indicators, Units of Measurement	ASTM D946-82 [29]		RK ST 1373 Requirements [31]			EN 12591 [34], Grade 70/100	Experimental Road Bitumen					
							Residue Above 480 °C		Residue Above 500 °C		Residue Above 525 °C	
	85–100	120–150	RCB 70/100	RCB 100/130	RCB 50/70		RCB 70/100	RCB 100/130	RCB 70/100	RCB 100/130	RCB 50/70	RCB 70/100
1. Needle penetration depth, 0.1 mm at 25 °C 0 °C, not lower	85–100 -	120–150 -	71–100 22	101–130 30	51–70 18	70–100 not rated	87 32	127 37	78 29	111 32	64 23	98 29
2. Softening temperature, °C, not lower	-	-	45	43	50	43–51	48.1	44.3	48.6	44.2	49.1	43.8
3. Extensibility, cm at least 25 °C 0 °C	100 -	100 -	75 3.8	90 4.0	65 3.5	not rated	67 3.5	78 3.8	96 4.1	114 5.2	121 4.8	143 6.0
4. Brittleness temperature, °C	-	-	not/ab −18	not/ab −20	-	−10	−21.7	−23.2	−20.1	−21.8	−19.2	−20.4
5. Flash point not lower, °C	232	218	230				261	256	268	259	278	271
6. Penetration index	-	-	from −1.0 to + 1.0			−1.5 + 0.7	−0.1	−0.3	−0.5	−0.8	−1.0	−1.1
After heating in a thin film at 163 °C for 5 h												
7. Weight loss not more than, % by weight	not rated		0.6	0.8	0.5	0.8	0.4	0.5	0.35	0.41	0.28	0.32
8. Change in softening temperature, °C	not rated		not more 5	not more 5	not more 5	not lower 45	4.0	4.2	3.8	4.0	3.3	3.8
9. Needle penetration depth at 25 °C, in % of initial	not rated 47	not rated 42	not rated	not less 50	not less 43	not less 46	68	64	71	69	78	72

## Data Availability

The original contributions presented in this study are included in the article. Further inquiries can be directed to the corresponding authors.
